# Learning Together: A Mixed Methods Study of Interprofessional Mental Health Simulation in Undergraduate Medical and Nursing Training

**DOI:** 10.7759/cureus.94239

**Published:** 2025-10-09

**Authors:** Thomas Davies, Sung Yeon Kwak, Laura Sevenoaks, Shreiya Narayanan, Chris Jacobs

**Affiliations:** 1 Urology, North Bristol NHS Trust, Bristol, GBR; 2 Medical Education, Great Western Hospital, Swindon, GBR; 3 Internal Medicine, Great Western Hospital, Swindon, GBR; 4 Medical Education, King's College London, London, GBR; 5 Psychology, University of Bath, Bath, GBR; 6 Medical Education, Great Western Hospitals, Swindon, GBR

**Keywords:** inter-professional collaboration skills, medical nursing, mental health services, simulation medicine, undergraduate medical student

## Abstract

Introduction:Interprofessional education (IPE) involves learners from two or more health professions creating a collaborative learning environment. With the growing use of simulation in medical education, interprofessional simulated learning (IPSL) has had increasing focus on its role in interdisciplinary collaboration, especially in the undergraduate setting.

Methods: This mixed methods study was conducted across two universities in South West England, incorporating Year 5 medical students and Year 3 nursing students. The IPSL experience was designed as three different simulation scenarios focusing on the assessment and management of mental health (MH) patients, with an emphasis on interprofessional collaboration. Study participants were asked to complete pre-session and post-session questionnaires with Likert scale and free-text questions used to collect the quantitative and qualitative data. The information collected related to students' confidence in working with MH patients and working with each other.

Results: Twenty-nine final-year healthcare students (19 nursing, 10 medical) from two universities participated in a single-day mental health interprofessional learning (IPL) simulation. Wilcoxon signed-rank tests demonstrated significant improvements from pre- to post-intervention for six of the seven items (all p ≤ 0.001), with median increases ranging from 0.5 to 1.5 Likert points. Qualitative data analysis generated four main themes: working collaboratively, understanding professional roles and skill sets, confidence in managing mental health problems, and improving communication.

Discussion: Our research aligns with current literature regarding the importance of IPE within healthcare. In particular, this study highlights the value of IPSL in increasing student confidence in working with other healthcare professionals and understanding their roles within the clinical setting. Further additional research is required to review the use of IPSL in undergraduate medical and nursing degrees and its role in evaluation of mental health conditions in adult medicine.

Conclusion: This study has shown that interprofessional learning through the form of mental health simulation can improve healthcare students’ confidence in managing acute MH crisis, understanding of professionals’ roles and collaborative working.

## Introduction

Interprofessional education (IPE) involves learners from two or more health professions creating a collaborative learning environment [1]. This method of learning is thought to facilitate the preparation of students to provide patient care in a collaborative environment [2]. The benefit of IPE not only lies in the exploration of team roles and attitudes but also in collaborative teamwork and improving communication between professionals [3]. The increasingly complex health needs of patients, requiring input from multiple disciplines and specialities, have highlighted the importance of IPE in healthcare curricula [4]. An increasingly prevalent method of IPE includes interprofessional simulated learning (IPSL) [5] to allow simulated experiences to facilitate student reflection on collaborative practice between different professionals [6].

Simulation is an educational technique where scenarios are designed to recreate events that occur in clinical practice [7]. It creates an environment where students can safely make mistakes and learn from them [8]. Simulation-based education has moved away from the traditional apprenticeship style of healthcare education and draws on constructivist learning theory. Constructivism is the idea that learners construct their own knowledge often through authentic tasks [9]. Simulation is being increasingly used within healthcare education [10] and has been associated with improved patient outcomes [11].

Not only has simulation-based education shown to improve communication and team working skills but also attitudes towards people with mental health [12]. Despite the discussed benefits of IPSL, much of this education is delivered in postgraduate education [13], and research has suggested that more exposure to IPSL should be provided in undergraduate medical education due to its positive impact on inter-disciplinary collaboration [14]. Our study aims to evaluate the impact of a mental health themed IPSL experience for medical, nursing, and allied health professional students. Our study focused not only on the clinical aspects of the simulation but also on the students’ attitudes towards interprofessional learning and collaboration between disciplines.

## Materials and methods

Study design and participants 

This mixed methods exploratory design study was conducted across two universities in South West England and incorporated fifth-year medical students and third-year nursing students. This quality improvement study was done in accordance with the UK Health Research Authority (HRA) guidelines. The HRA research criteria determine this study as a service evaluation; therefore, the NHS Research Ethics Committee (REC) was not required. All data collected was stored securely and anonymously.

Intervention

The IPSL experience was designed as three different simulation scenarios focusing on the assessment and management of mental health patients, with an emphasis on interprofessional collaboration. The simulations were created by a group of medical education specialists with nursing and medical backgrounds with the aid of a mental health expert. The simulations ran for 20 minutes and were followed by a 40-minute debrief co-led by a member of nursing faculty and a member of the medical faculty. The simulations covered the topics of eating disorder with electrolyte imbalance, self-harm, and elder abuse. Appendix 1 includes the scenarios used in this learning experience.

Data collection

Study participants were asked to complete a pre-session and post-session questionnaires with Likert scale and free-text questions used to collect the quantitative and qualitative data. The questionnaires were designed by the researchers and peer-reviewed by other medical doctors within the medical education field, and formal validation was not completed. The questionnaire allowed students to record their confidence in the underpinning clinical content of the simulation, as well as their understanding and views of the multidisciplinary team and their confidence working within them. Post-session questionnaires contained the same Likert scales, and qualitative feedback was collected about how valuable students found the session and how the interprofessional aspect affected their learning experience. The questionnaires used are included in Appendix 2.

Data analysis

Thematic analysis was conducted on the qualitative data using the six-step data analysis process of Clarke and Braun [15] to manually generate codes and themes (LS). This process was repeated by another researcher (TD). The researchers were both clinicians working in the field of medical education; therefore, it is important to highlight that the analysis would have been completed with this specific lens. The process involved familiarisation with the data, generation of initial codes, and searching and reviewing themes. The emerging themes were collaboratively generated through an iterative process. Disagreements were resolved through discussion until a consensus was reached (TD, LS). No formal analysis software was used.

Descriptive statistics were calculated using medians and interquartile ranges (IQR) for Likert-scale responses, as these ordinal data do not meet normality assumptions. The Wilcoxon signed-rank test was used to evaluate pre-post changes, appropriate for paired ordinal data with small sample sizes. Statistical significance was set at p<0.05. Effect sizes with values of 0.1, 0.3, and 0.5 indicating small, medium, and large effects were used. Subgroup comparisons between nursing and medical students were performed using Mann-Whitney U tests, although interpretation was limited by unequal group sizes.

## Results

Quantitative data analysis

**Figure 1:**
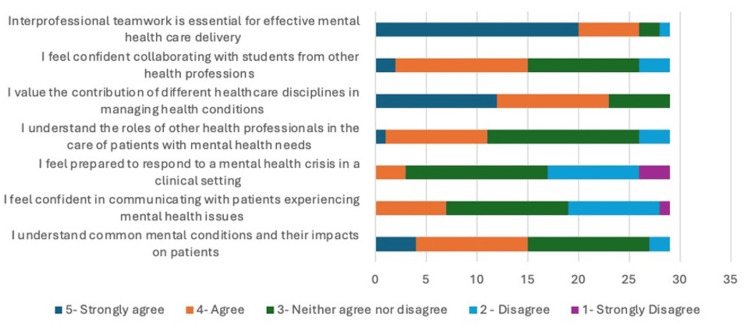
Pre-Session Questionnaire Summary in Likert Scales

**Figure 2:**
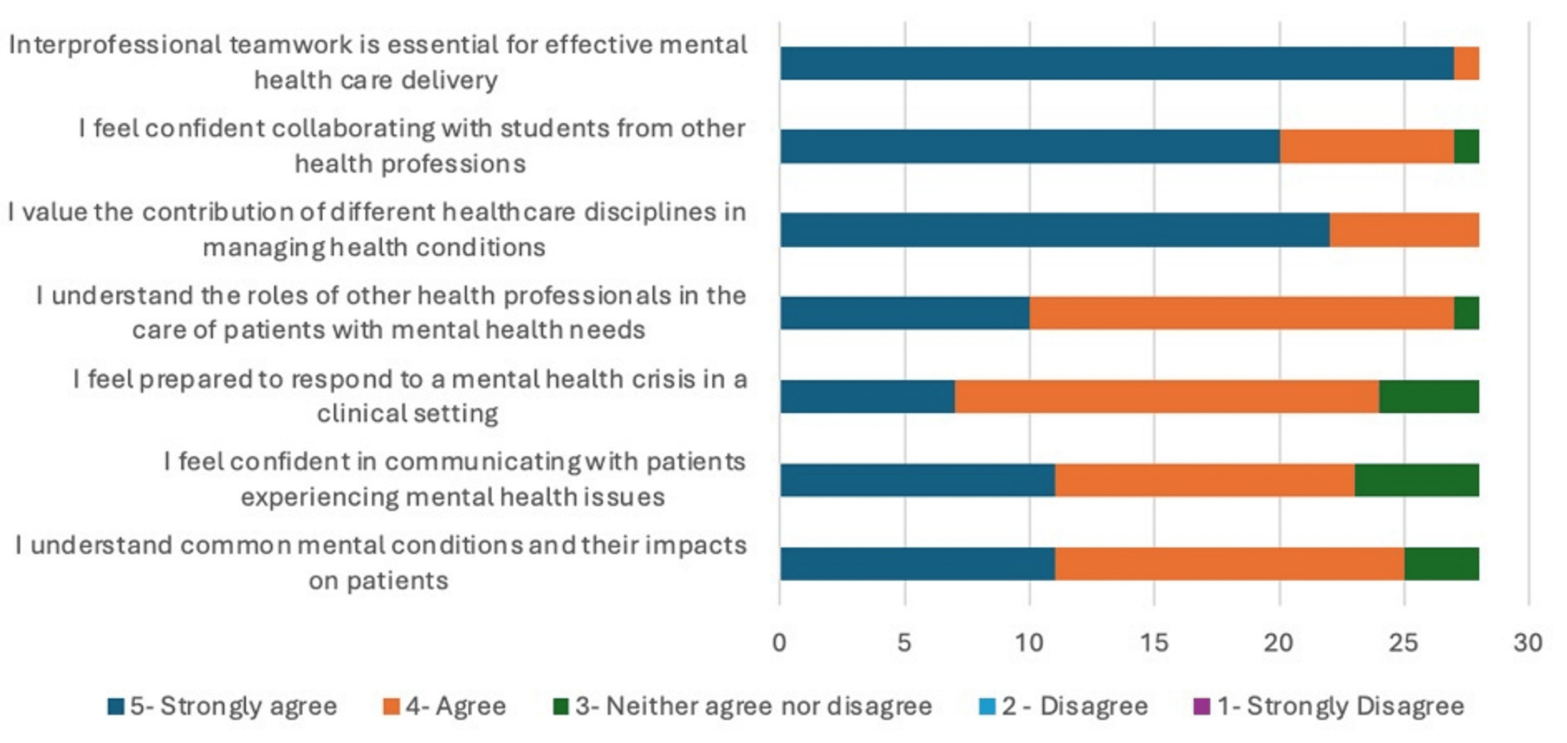
Post-Session Questionnaire Summary in Likert Scales

Twenty-nine final-year healthcare students (19 nursing, 10 medical) from two universities participated in a single-day mental health interprofessional learning (IPL) simulation. Complete paired pre-post data were collected from 28 participants (96.6% response rate), with one loss to follow-up. A summary of all the pre- and post-questionnaire findings is highlighted in Appendix 3. An infographic of these findings can be seen in Figures 1, 2.

In the pre-session questionnaire, 20 students (71.4%) strongly agreed that interprofessional team is essential for effective mental healthcare delivery. Following the session, this increased to 27 (96.4%). Prior to the session, 12 students (42.8%) strongly agreed with the statement "I value the contribution of different healthcare disciplines in managing mental health conditions," this increased to 22 students (78.6%) following the simulation.

Our results showed seven students (25%) agreed or strongly agreed that they felt confident collaborating with students from other health professions pre-session, this increased to 23 students (82.1%) post-session. There was also an increase seen in the number of students who agreed or strongly agreed with the statement "I understand the roles of other health professionals in the care of patients with mental health needs" from 11 (39.2%) to 27 (96.4%).

There was also a notable improvement in the student’s confidence with managing mental health crisis following the simulation. Only three students (10.7%) agreed or strongly agreed with the statement "I feel prepared to respond to a mental health crisis in a clinical setting prior to the simulation." When comparing this to the post-session feedback, 24 (85.7%) agreed or strongly agreed. Our results demonstrated an increase from seven students (25%) who agreed or strongly agreed with the statement "I feel confident in communicating with patients experiencing mental health issues" pre-session to 24 (85.7%) post-session. When asked, 15 students (53.5%) agreed or strongly agreed with the statement "I understand common mental conditions and their impacts on patients" prior to the session which increased to 24 students (85.7%) post-session.

Twenty-nine participants (nursing = 19, medicine = 10) completed both pre- and post-intervention questionnaires. Rank-based regression models were used to examine the effect of programme (medicine vs nursing) and year of study on post-intervention Likert scores. Table 1 provides a summary of these results.

**Table 1:** Wilcoxon Signed-Rank Test Results Data

Item	n	Median (Pre)	Median (Post)	Median change	Effect size (r)	p
I understand common mental conditions and their impacts on patients	28	3.5	4.0	0.5	0.62	0.001
I feel confident in communicating with patients experiencing mental health issues	28	3.0	4.0	1.0	0.75	<0.001
I feel prepared to respond to a mental health crisis in a clinical setting	28	3.0	4.0	1.0	0.83	<0.001
I understand the roles of other health professionals in the care of patients with mental health needs	28	3.0	4.0	1.0	0.73	<0.001
I value the contribution of different healthcare disciplines in managing health conditions	28	4.0	5.0	1.0	0.61	0.001
I feel confident collaborating with students from other health professions	28	3.5	5.0	1.5	0.78	<0.001
Interprofessional teamwork is essential for effective mental healthcare delivery	28	5.0	5.0	0.0	0.47	0.013

Wilcoxon signed-rank tests demonstrated significant improvements from pre- to post-intervention for six of the seven items (all p ≤ 0.001), with median increases ranging from 0.5 to 1.5 Likert points. The largest median increase was observed for “I feel confident collaborating with students from other health professions” (median change = 1.5, p < 0.001), followed by “I feel prepared to respond to a mental health crisis in a clinical setting” and “I understand the roles of other health professionals in the care of patients with mental health needs” (both median change = 1.0, p < 0.001). “Interprofessional teamwork is essential for effective mental healthcare delivery” showed minimal change (median change = 0.0) but remained statistically significant (p = 0.013), likely due to a high pre-intervention median of 5.0. Effect sizes (r) ranged from 0.47 to 0.83, indicating moderate to large magnitudes of change.

Year of study was a consistent positive predictor of higher post-intervention scores across all items (β range = 2.65-3.27, all p < 0.001), indicating that students in later years reported greater perceived competence and confidence. Full regression results are seen in Table 2.

**Table 2:** Full Regression Results

Item	Beta programme	95% CI (programme)	p_programme	Beta year	95% CI (year)	p_year
I understand common mental conditions and their impacts on patients	-9.38	-14.69 to -4.08	0.001	2.86	2.14 to 3.59	<0.001
I feel confident in communicating with patients experiencing mental health issues	-12.15	-17.19 to -7.11	<0.001	2.65	1.96 to 3.34	<0.001
I feel prepared to respond to a mental health crisis in a clinical setting	-9.45	-14.55 to -4.35	<0.001	2.86	2.16 to 3.56	<0.001
I understand the roles of other health professionals in the care of patients with mental health needs	-5.0	-10.16 to 0.16	0.057	3.2	2.49 to 3.91	<0.001
I value the contribution of different healthcare disciplines in managing health conditions	-4.06	-8.27 to 0.16	0.059	3.27	2.69 to 3.85	<0.001
I feel confident collaborating with students from other health professions	-6.82	-11.59 to -2.06	0.007	3.06	2.41 to 3.71	<0.001
Interprofessional teamwork is essential for effective mental healthcare delivery	-5.54	-7.47 to -3.61	<0.001	3.16	2.89 to 3.42	<0.001

Programme was a significant predictor for five of the seven items, with medicine students scoring lower than nursing students after adjusting for year of study. For example, medicine students reported lower confidence in communicating with patients experiencing mental health issues (β = -12.15, 95% CI = -17.19 to -7.11, p < 0.001) and in responding to a mental health crisis in a clinical setting (β = -9.45, 95% CI = -14.55 to -4.35, p < 0.001). No statistically significant programme effect was observed for understanding the roles of other health professionals (p = 0.057) or valuing the contribution of different healthcare disciplines (p = 0.059).

Qualitative data analysis

The post-session questionnaire asked the students what they found most valuable about this simulation and four major themes emerged from the codes. Table 3 provides a summary of these four main themes.

**Table 3:** Qualitative Data Themes With Core Quotations MH: mental health, MDT: multiple disciplinary team.

Theme	Quotation number	Quotation	Participant
Working collaboratively	1	“Understanding more about how to work with doctors ”	P9
	2	“Collaboration between the team”	P23
	3	“Having a chance to work as an MDT with the Drs”	P26
	4	“Importance of collaboration”	P5
Understanding professional roles and skill sets	5	“More aware of others roles”	P4
	6	“Learning what nurses roles are”	P5
	7	“Different skill set of the nursing students”	P11
	8	“Understanding different roles in varying situations”	P16
Confidence in managing mental health patients	9	“Understanding how to look after patients with MH issues”	P9
	10	“How to deal with common MH issues”	P7
Improving communication	11	“The importance of clear communication”	P13
	12	“Learning to be more confident in asking difficult questions”	P1

Working Collaboratively

This theme emerged from the following codes: working together and learning about the value of collaboration. Students repeatedly highlighted the value of working with other professionals and working as a multidisciplinary team which is keeping with real practice on the wards and in many clinical environments (quotes 1-4).

Understanding Professional Roles and Skill Sets

This theme comprised the following codes: learning about each other’s roles and learning how to split tasks. Both codes were commonly found in student responses about the valuable aspects of the simulation. The code learning about each other’s roles featured more heavily in the medical student’s responses compared to nursing students. Further, medical students appeared to appreciate nursing students' roles and responsibilities (quotes 5-8).

Confidence in Managing Mental Health Patients

This theme originated from the codes, confidence in mental health management, and understanding the scope of mental health presentations. Students highlighted the value of learning how to manage common mental health presentations and how to care for patients experiencing mental health crisis in their responses (quotes 9-10). This theme is also reflected in the students' increased confidence when rating their ability to manage mental health presentations in the pre- and post-session questionnaire.

Improving Communication

This theme encompassed the valuable learning about the importance of clear communication with each other and with the patients. Students found that the simulation provided an opportunity to become more confident in asking questions they perceived as more difficult, and they acknowledged the value in learning how to communicate effectively in situations specific to mental health presentations (quotes 11-12).

## Discussion

This mixed methods study aimed to review the role of mental health interprofessional simulations in undergraduate nursing and medical students from two different universities. Following the simulations, the students reported substantial increases in confidence for managing mental health patients, working with other healthcare students and managing those in acute MH crisis. In addition, there was an increase in appreciating the need for an interprofessional approach when managing mental health patients and understanding the different, applicable roles in these circumstances. The qualitative analysis revealed four main themes: working collaboratively, understanding professional roles and skill sets, increasing confidence in managing MH patients, and improving communication.

Our research aligns with current literature regarding the importance of IPE within healthcare. In particular, this study highlights the value of IPSL in increasing student confidence in working with other healthcare professionals and understanding their roles within the clinical setting. This study adds to the importance of maintaining IPSL within the medical curricula to ensure that healthcare professionals work effectively together [16]. This is also additionally supported by the World Health Organisation (WHO) who highlighted that teaching within a multi-professional setting in student years can increase effective teamwork between healthcare professionals [17].

On completion of a scoping review, only one study by Attoe et al. [18] has looked at the role of mental health simulations in the early stages of professional training. The study emphasises the value of IPSL within undergraduate healthcare students at both an individual and professional level in hope that there would be longer-term benefits seen in clinical care delivered by the participants. Although we have not directly studied the potential future benefits of including IPSL at an undergraduate level, the qualitative data suggests an increasing appreciation for working collaboratively with other healthcare members and an increasing confidence within a mental health setting. Therefore, it is suggestive that the use of these learning exercises in undergraduates is likely to change and adapt patient clinical care in the future, although this is difficult to measure in the long term.

The scenarios used in this study were created in clinical environments such as the emergency department and general practice. This was designed in order to provide overlap between mental health and physical health in patients. All the nursing students involved were adult nursing students, and therefore, placing them in these clinical settings facilitated increased psychological and sociological fidelity. There is evidence to suggest that close alignment with their current roles facilitates important practice change [19].

Limitations

Several limitations are to be acknowledged. The study includes a small sample size of 29 students from two different universities participating in an interprofessional, interuniversity simulation. Therefore, the data is not representative of all UK universities, and we do not know how this study fits within the national context of undergraduate education. The post-session questionnaire was completed immediately following the scenario, and there has not been any long-term follow-up of the students to assess the impact of the IPLS after graduation or how it has affected their clinical work and attitudes.

A further limitation to highlight is the use of independently developed questionnaires in this study. They had not undergone formal validation for reliability or construct validity. Hence, there is a potential for underlying assumptions and researcher bias when completing the thematic analysis.

Future research

Further additional research is required to review the use of IPSL in undergraduate medical and nursing degrees and its role in evaluation of mental health conditions in adult medicine. The differences and impact of its use in undergraduate education should be compared to its use in postgraduate education and its role in both curricula. The timing of introduction into undergraduate education should also be considered in further research.

## Conclusions

This study has shown that interprofessional learning through the form of mental health simulation can improve healthcare students’ confidence in managing acute MH crisis, understanding of professionals’ roles, and collaborative working. These findings align with existing literature and further reinforce the value of interprofessional learning to prepare students for clinical environments and working within a multiple disciplinary team (MDT). This area would benefit from further research evaluating the long-term impact of incorporating interprofessional simulation into medical and nursing curricula.

## Figures and Tables

**Figure 3:**
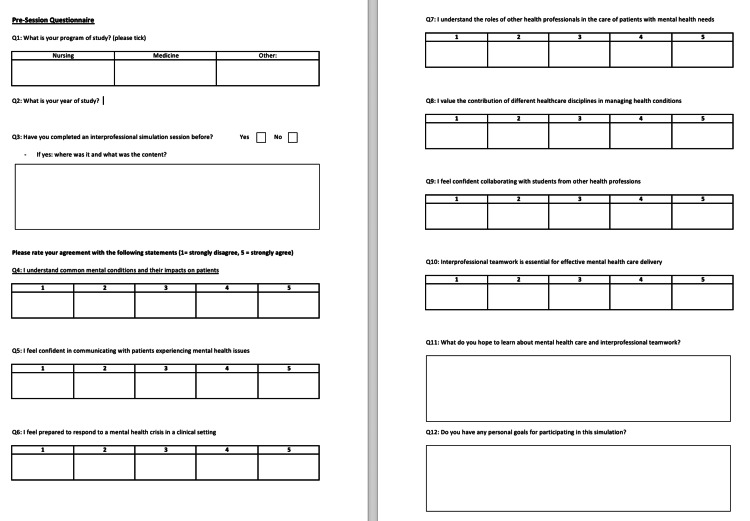
Pre-Session Questionnaire

**Figure 4:**
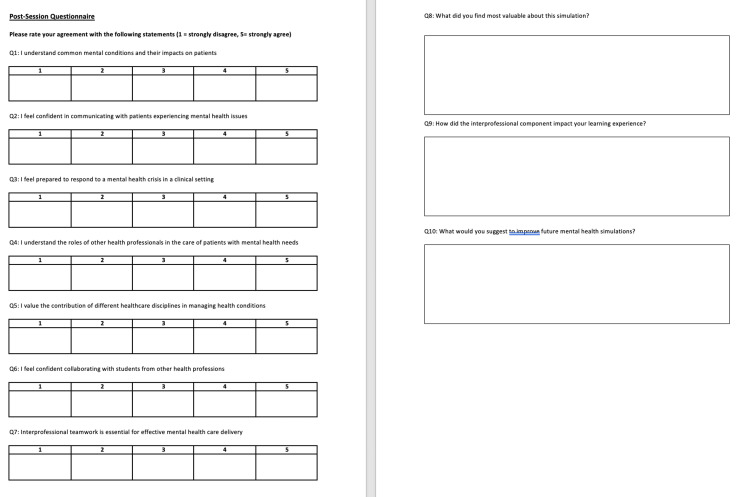
Post-Session Questionnaire

## Appendices

Appendix 1: Interprofessional learning scenarios

**Table 4:** Scenario 1: Crisis With Suicidal Ideation F1: Foundation Year 1, GWH: Great Western Hospital, CTF: Clinical Teaching Fellow, OBU: Oxford Brookes University, A-E: medical assessment, NEWS2: New Early Warning Score, PRN: as required, SMOTS: Scotia Medial Observation and Training System.

Scenario overview	
Scenario synopsis	Jamie Taylor is a 30-year-old individual who was brought to the Emergency Department (ED) following self-harm. Jamie was discovered at home by their housemate, who noticed Jamie appeared withdrawn and upset for several days and found their shirt sleeve to be blood soaked. The housemate immediately called for help, and Jamie was brought into ED by ambulance. Upon arrival at ED, Jamie was tearful and agitated about being brought in. Initial assessment by the nursing staff revealed that Jamie had multiple superficial lacerations on their left wrist extending to their elbow, which Jamie was reluctant to show and receive treatment for; they remained teary and quiet during the assessment and are reluctant to make eye contact with the healthcare team. Jamie has a history of low mood but has not sought any professional help and has no previous psychiatric admissions. They are currently not under the care of any mental health services. The nursing staff managed to establish a rapport with Jamie, prompting them to disclose more about their feelings and the circumstances surrounding the self-harm. Jamie revealed that they had been struggling with increased isolation and stress due to recent job loss and the end of a significant relationship in the last two months. During the assessment, Jamie mentioned having a supportive older sister, Alex, who lives nearby. Jamie has not felt like themself lately and has stopped attending social activities they once enjoyed. Jamie also used to have a passion for gardening which they used to do with their late grandmother. They share fond memories of gardening with their late grandmother, particularly growing roses, which Jamie cultivates in their garden as a way to feel connected to her. Over the last two months, they have not been out in the garden and let it come to disarray. As the nursing staff continued their evaluation, Jamie's reluctance to engage with the doctors became evident, leading to a discussion on the importance of creating a safe and trusting environment for them to share their concerns. The ED team will need to manage both the medical aspects of wound care and the critical mental health assessment, as well as involve psychiatric services for further support and intervention.
Learner participants and learner level	Adult nurses year 3/medical students/F1 GWH
Scenario authors and content expertise	Dr Shreiya Narayanan, CTF, GWH; Dr Laura Sevenoaks, CTF, GWH; Dr Sung Yeon Kwak, CTF, GWH; Helen Krawczyk, Lecturer, Adult Nursing, OBU; Janice Watson, Senior Lecturer, Simulation and Immersive Technology, OBU; Dr Jon Freeman, Clinical Lead and Psychologist, GWH; Dr Karen Lascelles
Up-to-date key references for scenario content	https://www.nice.org.uk/guidance/ng225; https://www.ucl.ac.uk/pals/research/clinical-educational-and-health-psychology/research-groups/competence-frameworks/self-harm; Wound care management and assessment, managing of self-harm wounds
Estimated scenario time	15-20 minutes
Estimated debrief time	30-40 minutes
Curriculum/module/scenario learning outcomes	
Learning objectives	Knowledge: Able to identify the signs and presenting symptoms of a patient presenting with self-harm wounds; prioritises and initiates appropriate interventions for a patient who presents with self-harm wounds; and identifies concerns regarding instruments used to self-harm and considers wider implications of vaccination status of patient. Skills: Demonstrates the ability to establish rapport and communicate empathetically with a distressed patient, employing active listening and de-escalation techniques; and demonstrates an effective handover of clinical information using a structured format (Situation Background Assessment Recommendation (SBARR)). Attitudes/behaviours: Demonstrates effective communication and teamwork between different professional groups; and demonstrates effective timely communication, empathy, support, and active listening with the patient and family if present.
Prerequisite knowledge required and/or pre-session work?	Management of superficial laceration. How to take a mental health history? How to assess risk? Knowledge of referral pathways.
Scenario design	
Scenario environment	Location/setting: Emergency Department (ED). Patient: Actor/faculty acting as patient (Jamie Taylor). Monitor: Yes (required to assess vital signs). Props/equipment/medical Supplies: ED stretcher. Patient table. Patient chair positioned beside the bed. Blood pressure cuff, pulse oximeter machine. Thermometer Armband. A-E Trolley including nasal cannula/masks, needles, syringes, blood bottles and gas syringes. IV fluids various, giving sets syringe for fluid administration and medication administration (0.9% NaCl 100 ml and 500 ml bag). Capillary blood glucose/ketone meter. Intramuscular tetanus syringe 1 ml of 250 IU tetanus immunoglobulin (TIG). Tablets for oral Co-amoxiclav 250 mg/125 mg (Label Amoxicillin (as Amoxicillin trihydrate) 250 mg, Clavulanic acid (as Potassium clavulanate) 125 mg). Wound dressing pack: Sterile gloves, one pack of each size. Gauze: Several packs, Normosol, Mepore dressing (sufficient to cover wound/s). Clipboard with initial triage notes. Observation chart (NEWS2). Pens and patient notes. Make-up/moulage/dress: Jamie is dressed in casual clothes (e.g., jeans and a t-shirt) that look slightly rumpled, as if they have been wearing them for an extended period. Dressed in full sleeves with blood-stained left sleeve, four to five new lacerations with dried blood on left forearm; with old, healed lacerations in between. Also two older lacerations looking a little red and inflamed. Jamie should appear emotionally distressed: tear stains or light smudges of make-up, if applicable. Jamie wears a wristband with their name and allergy information (if applicable). Multimedia/paperwork: Patient observation chart (NEWS2 chart). Types of wounds and their management poster, if applicable. Medication chart. Assessment tool: Mental Health Questionnaire from GWH in drive. Audio/visual support: Simulated phone for escalation to the psychiatric liaison team or senior ED physician Scotia Medical Observations and Training System (SMOTS) camera or equivalent for live observation of the scenario. People: Lead sim faculty (facilitator guiding the simulation). Actor as the patient (Jamie Taylor). Nursing student actors (perform initial assessment). Medical student actors (called after initial nursing assessment). Healthcare assistant (HCA) hands over to nursing student and expresses their concerns. Faculty/plant as ED psychiatrist or senior doctor to answer phone and provide guidance if escalated.
Patient profile clinically realistic and summarised with detail regarding their characteristics, history and current status	Demographics: Name: Jamie Taylor. Age: 30 years. 02/04. Address: Flat 1, 231 Bond Street, Swindon, SN1 1BT. Weight: 60 kg. Height: 5’7”. Past medical history (PMH): Eczema (since childhood, managed with topical treatments); mild asthma (diagnosed in adolescence, occasional use of inhaler, no recent exacerbations); no prior hospital admissions for significant medical issues; and unsure of their vaccination history. Family history (FH): Mother: History of eczema, alive and well, living locally; Father: Died in a road traffic accident when Jamie was 12; and Grandmother (maternal): Passed away from lung cancer, shared a close relationship with Jamie; and no known family history of asthma or significant respiratory conditions. Social history (SH): Currently lives with a roommate in a shared ground floor apartment. Recently lost their job at a local café two months ago, which has contributed to feelings of isolation and stress. Broke up with a long-term partner one months ago, which has added to their emotional strain. Jamie enjoys gardening, particularly growing roses, a hobby they took up in memory of their late grandmother. They have an older sister, Alex, who lives nearby and is a significant support figure. Jamie has not been in contact with mental health services and has no prior history of psychiatric conditions or treatments. Drug history (DH): Salbutamol Inhaler 100 µg prn (used occasionally for asthma, last used one week ago). Emollients for eczema (used as needed). Paracetamol 1 g prn (used occasionally for headaches). No other regular medications. Allergies: None known.
Initial Sim man setup or actor information for faculty	Provide all information here on initial settings required for your scenario. Jamie self-harmed approximately two hours before being brought into ED. They used an old blade they found to make lacerations along skin and did not sterilise the instrument. They have not dressed the wound or cleaned it. Emotionally distressed, tearful, and feeling hopeless. Initially cooperative with nursing staff but guarded with doctors. Expressed vague suicidal thoughts, mentioning that they "didn't want to be here anymore" but not providing specifics. Vital signs are stable, but Jamie’s mental state is fragile. They appear emotionally withdrawn, not making eye contact but become more responsive to empathetic, non-judgmental communication.
Actor brief	Upon arrival of nursing staff: You are sitting on the edge of the hospital bed, looking distressed. You are in pain following self-harm in your left forearm. Initially, you are cooperative with the nurses, but struggle to maintain eye contact. You disclose that you self-harmed with an old blade, but you feel like you don’t want help. If asked about how you’re feeling emotionally, you open up a little. Mention feeling isolated, not having the energy to do things you once loved (like gardening), and talk briefly about your breakup. However, don’t fully articulate your suicidal thoughts unless they really try to understand you. You’re scared but also feel like you don't want to be a burden, so you minimise some of your symptoms or say, "I’m fine, it’s not a big deal." When doctor arrives: You become guarded, not trusting them as much as the nurses. You give short answers and avoid eye contact. You tell them you “didn’t mean to self-harm” downplaying the incident. If they press for details, you get irritated and may say something like, "Why does it even matter? It’s not like anybody actually cares." If they engage you in personal conversation: If they ask about your family or hobbies, such as your gardening or your sister Alex, you slowly warm up to them. Mention how much you enjoyed growing roses with your grandmother and how you haven’t been able to care for them lately. If they mention your sister Alex, acknowledge that she tries to be supportive but you feel guilty for leaning on her too much. If they attempt to assess your mental health: If the healthcare professionals ask directly about suicide, initially deflect the question with, “It’s not important,” or “I don’t want to talk about that.” and become tearful. If they handle the situation calmly and show genuine concern, you can admit, “I didn’t want to be here anymore,” or say something vague like, “I just thought it would be easier if I wasn’t around.” De-escalation response: If they handle the situation with empathy and understanding, you begin to calm down. You might start to share more about how difficult things have been recently and eventually admit you feel scared about what you've done. If they don’t engage with you on a personal level, you remain withdrawn, defensive, and agitated. Ending the scenario: The scenario should end when the healthcare professionals establish rapport, and you feel comfortable enough to lie down on the bed, begin receiving treatment for the wound and allow them to involve mental health services. Allow them to take the lead in the next steps of your care if they’ve shown care and concern.
Student pre-brief psychological safety, learning outcomes, ground rules, orientation to simulation environment, students expectations, all given in group pre-simulation pre-brief	To all: Jamie Taylor is a 30-year-old individual who was brought to the Emergency Department (ED) following self-harm. Jamie was discovered at home by their housemate, who noticed Jamie appeared withdrawn and upset for several days and found their shirt sleeve to be blood soaked. The housemate immediately called for help, and Jamie was brought into ED by ambulance. Scenario pre-brief: Nursing student: You are a newly qualified nurse working in the Emergency Department (ED). You are going to assess Jamie Taylor, a 30-year-old patient who has presented to the ED emotionally distressed. The healthcare assistant (HCA) is with Jamie and will hand over to you. Scenario pre-brief: Medical Student: You are an F1 doctor working in the Emergency Department (ED). You have been called by the nursing staff to review Jamie Taylor, a 30-year-old patient who presented to ED with emotional distress.
HCA brief	You are the healthcare assistant (HCA) working in the Emergency Department (ED). You have been trying to manage Jamie Taylor, the patient, who is emotionally distressed and withdrawn. Jamie has not yet disclosed to anyone about any injuries sustained, but you have noticed their blood-stained sleeve and are concerned about their behaviour. You have only been able to gather minimal information from them. You have managed to just complete a set of observations which you have recorded on the NEWS2 chart. Scenario set-up: When the nursing staff arrives, greet them by explaining that Jamie has been "quiet and hard to engage" today. You haven’t been able to get much information from them. Starting position: Jamie may be sitting quietly or pacing, visibly upset or withdrawn. You are in the room, having just tried to talk to Jamie, but they have given you very little response. You could say something like, “I’ve tried asking Jamie what’s wrong, but they just won’t open up to me.” Your role: Jamie has not disclosed to you about the self-harm, so you don’t know any specifics about what has happened. However, you sense that something is wrong because Jamie seems detached and has a blood-stained sleeve. You’ve asked the nursing staff to come and assist because you’ve found it difficult to get Jamie to engage in any meaningful conversation or share any relevant details about their condition. During the scenario: When the nurses arrive, give them a summary of your interaction with Jamie: “Jamie has been really quiet and hasn’t told me much. I’ve been trying to get them to talk, but I’m worried about them." “I have just managed to do a set of vitals which I have recorded on the NEWS2 chart.” Encourage the nursing staff to take over and try to engage Jamie: “Maybe you can get through to them, I think they just don’t want to talk to me.” After handing over, step out of the room to give the nurses space to assess Jamie on their own, but let them know you’ll be available if they need you: “I’ll step out for a moment, but let me know if you need anything.” Key phrases to use: “They’ve been really withdrawn, I couldn’t get them to say much.” “Jamie seems upset, but I haven’t been able to get through to them.” “Maybe they’ll open up to you.”
Participant roles	x2 Student nurses assume the role of a newly qualified graduate, x1 Medical students assume the role of an F1
Observer roles	Communication: Therapeutic relationship building, handover information (SBARR), assessment skills, teamworking
Faculty information appropriate suggestions are incorporated to increase or decrease the scenario complexity	None
Debrief	
Plan for debrief	Use pause technique followed by shorter debrief co-debrief: Nursing Faculty and Lead Medical Faculty
Debrief	Which debrief tool will you use? Debriefing questions you might want to help structure the debrief. Diamond Tool (Examples: Give a brief summary of what happened? What were the challenges? What worked well? If you were to do this again, how would you handle the situation differently?) Space for notes to make to bring up in debrief, key learning points. How to identify and address key performance gaps. Review of learning outcomes. How will observers be incorporated? Observers on SMOTS with areas to observe: Communication, handover information (SBARR), assessment skills/teamworking.
Post-simulation learning activities (see some examples below)	
Reflection	Identify any reflective activity and requirements
Skills lab	Follow-up skills lab work
Goal setting	Participants develop goals for future development
Self-assessment activity	Participants may do a post self-assessment activity
Quiz	Develop a quiz post-simulation to identify learning and achievement of learning outcomes

**Table 5:** Scenario 2: Electrolyte Disturbance and Eating Disorder GWH: Great Western Hospital, CTF: Clinical Teaching Fellow, OBU: Oxford Brookes University, A-E: medical assessment, NEWS2: New Early Warning Score, SMOTS: Scotia Medial Observation and Training System, BNF: British National Formulary.

Scenario overview	
Scenario synopsis	Eddie Greene is a 20-year-old physiotherapy student in their first week of placement at GWH. They have been brought to the Emergency Department (ED) by their classmate after they fainted on their ward during their placement. Eddie insists that it was a simple case of low blood sugar and that they haven't been eating well due to food intolerances. However, their flatmate has grown increasingly worried about Eddie, noting that they have been avoiding meals, staying in their room most of the time, and has lost a noticeable amount of weight over the past few months. Eddie presents as slightly pale and fatigued but is trying to downplay the severity of their symptoms. They have no known medical conditions recorded in their medical notes but claim to have multiple food intolerances, though they cannot clearly specify which foods affect them. When asked about their diet, they mention that they have been cutting out various foods to "feel better" and avoid discomfort but cannot provide a clear reason. They deny any history of eating disorders or mental health issues and insist that their busy academic schedule has been the primary cause of their missed meals. Eddie’s family lives out of town, and they have not been home often since starting university. They are unaware of Eddie’s weight loss or their eating patterns, as they rarely discuss their health with them. Their academic workload has become overwhelming, and they often use it as a reason to isolate themself from friends and social activities. Eddie has had no previous hospital admissions or significant medical concerns, but their flatmate is concerned that they are not telling the full story about their health. On examination in the Emergency Department (ED), their vital signs are concerning: their blood pressure is low, they are bradycardic, and appear to be dehydrated. Blood tests reveal an electrolyte imbalance, likely caused by poor nutrition. As Eddie continues to insist that their dietary choices are due to intolerances, the healthcare team must navigate their reluctance to disclose any deeper issues, while addressing the immediate medical concern of their electrolyte disturbance. Eddie has no formal diagnosis of an eating disorder, but their symptoms, behaviours, and the results of their tests raise suspicions of an undiagnosed restrictive eating pattern. The healthcare team must work together to uncover the underlying issue, offer supportive care, and initiate appropriate treatment while respecting Eddie's autonomy and encouraging them to open up about their health concerns.
Learner participants and learner level	Student, nurses in third year and final-year medical student, or Foundation Doctor (F1)
Scenario authors and content expertise	Dr Shreiya Narayanan, CTF, GWH; Dr Laura Sevenoaks, CTF, GWH; Dr Sung Yeon Kwak, CTF, GWH; Helen Krawczyk, Lecturer, Adult Nursing, OBU; Janice Watson, Senior Lecturer, Simulation and Immersive Technology, OBU; and Dr Jon Freeman, Clinical Lead and Psychologist, GWH.
Up-to-date key references for scenario content	Management hyponatraemia. Mental capacity act: https://www.nhs.uk/conditions/social-care-and-support-guide/making-decisions-for-someone-else/mental-capacity-act/. Eating disorders: Recognition and Treatment (NICE): https://www.nice.org.uk/guidance/ng69
Estimated scenario time	15-20 minutes
Estimated debrief time	30-45 minutes
Curriculum/module/scenario learning outcomes	
Learning objectives	Knowledge: Demonstrates an understanding of the medical complications of eating disorders, including the impact of prolonged poor nutrition on electrolyte balance and cardiovascular health; and prioritises and initiates appropriate interventions for a patient who presents with electrolyte imbalance suspected of having an eating disorder with reference to national guidelines. Skills: Undertakes a comprehensive assessment and demonstrates an effective A-E approach to patient assessment; demonstrates the ability to establish rapport and communicate empathetically with a distressed patient, employing active listening and de-escalation techniques; and demonstrates an effective handover of clinical information using a structured format (Situation Background Assessment Recommendation (SBAR)). Attitudes/behaviours: Demonstrates effective communication and teamwork between different professional groups; and demonstrates effective timely communication, empathy, support, and active listening with the patient and family if present.
Prerequisite knowledge required and/or pre-session work?	Management of electrolyte imbalances. How to take a mental health history? Knowledge of anorexia nervosa, i.e., presentation and key features. Referral pathway for eating disorder admission. Psychosocial impact of mental health and associated physical manifestations.
Scenario design	
Scenario environment	Location/setting: Emergency Department (ED). Patient: Actor. Monitor: Yes (to show observations). Props/equipment/medical supplies: Patient table; Plinth, with linen BP with SATS machine; thermometer; IV stand; and cannulation pad with pink cannula to be applied when appropriate which can be infused. Armband, ECG machine, spare trolley, and tray for med prep A-E trolley with nasal cannula/masks, needles, syringes, blood bottles, and gas syringes. IV fluids various, to include 0.9 NaCl, both with and without potassium available, giving sets syringe for fluid administration and medication administration Capillary blood glucose/ketone meter. Make up/moulage/dress: Age-appropriate clothing: Physiotherapy uniform baggy top and trousers. Pale. Multimedia/paperwork: Medication chart observations NEWS2 chart. Fluid balance chart ECG showing T wave inversion. Blood work results: BNF. Audio/visual support: SMOTS, telephone, headset to HCA/plant, and walkie talkies x2. Patient monitor for observations: People: Lead sim faculty (facilitator guiding the simulation). Actor as the patient: Emma Greene; Nursing student actors: perform initial assessment; and medical student actors (called after initial nursing assessment). Healthcare assistant (HCA) hands over to nursing student and expresses their concerns. Faculty/plant as ED physician or senior doctor to answer phone and provide guidance if escalated.
Patient profile Clinically realistic and summarised with detail regarding their characteristics, history and current status	Demographics: Name: Eddie Greene. Age: 20 years. Address: 14 Maple Lane, Oxford, OX3 7BT. Weight: 46 kg (recent weight loss). Height: 5’5”. Past medical history (PMH): Eczema (since childhood, managed with emollients). Family history (FH): Father: History of hypertension; Mother: History of anxiety, No known family history of eating disorders. Social history (SH): University student in their first year studying physiotherapy. Has become reclusive, spends long hours studying, and rarely socialises with friends or goes home to visit family. Eddie mentions they have been "very busy" with coursework and often skips meals due to "food intolerances and allergies," although these are not supported by their medical records. Has lost a noticeable amount of weight but has hidden this from their family by avoiding frequent visits home. Smokes occasionally, drinks alcohol occasionally in social settings. Drug history (DH): Emollients for eczema. Allergies: None known (despite the patient’s claim of food intolerances and allergies).Current presentation: Presents to the ED following a fainting episode at university accommodation. Denies any significant medical history beyond eczema, attributing their fainting to "stress and not eating properly" due to intolerances. Attempts to deflect questions about weight loss or eating habits. Appears anxious and reluctant to discuss personal habits in depth.
Initial Sim man setup or actor information for faculty	Provide all information here on initial settings required for your scenario. Eddie Greene is a 20-year-old university student studying physiotherapy who fainted on the ward due to an undiagnosed eating disorder. They are experiencing fatigue and mild dizziness but are hesitant to disclose the full details of their eating habits to medical staff. Emotionally distressed, they appear anxious and withdrawn, initially cooperative with the nursing staff but become defensive when questioned about their health. Vital signs may indicate mild dehydration, with possible hypotension and bradycardia. Eddie expresses vague concerns about their weight and dietary restrictions, mentioning allergies that are not documented in their medical history. They respond better to empathetic and non-judgmental communication, though may become agitated if their eating behaviours are challenged.
Actor brief	Character background: You are Eddie Greene, a 20-year-old university student studying Physiotherapy. Recently, you have become withdrawn and isolated, having stopped attending social events and avoiding interactions with peers. You fainted on the ward during your placement, and you have now been brought to the emergency department for assessment by your fellow physiotherapy student. Current state: You appear anxious and weak, reflecting your recent struggles with food intake and social interactions. You will exhibit signs of distress when healthcare professionals approach you and may resist their attempts to help. Key behaviours: Initial presentation: Start by looking pale and shaky, possibly sitting on the bed with your arms crossed, avoiding eye contact with the staff. Evasive responses: When asked about your diet or health, respond with vague excuses about being too busy with academic work. State that you are "fine" or "just stressed" to deflect attention from your eating habits. Resistance to care: If healthcare staff try to discuss your fainting episode, avoid answering directly. “I have had a couple of fainting episodes at home over the past few months when I stand up too quickly, and I have always been fine. Nothing to worry about” If asked about what you last ate, say something like, “I didn’t have time to eat last night; I was studying,” or “I usually skip breakfast.” Emotional fluctuation: As the staff engages with you, alternate between appearing anxious and then briefly calming down when they show understanding. However, remain defensive, especially if they try to encourage you to eat or drink. Distrust: If offered food or drink, express scepticism, saying things like, “I don’t know if I can eat that,” or “What’s in it? I have food intolerances.” Accept a sealed water bottle or snack, but only if it’s clearly labelled as safe for you. Avoiding assistance: If asked to sit down or relax, respond with reluctance, saying, “I need to keep moving,” or “I just want to get back to my studies.” You may start to pace slightly, showing your anxiety. Expressing isolation: Mention feeling overwhelmed by your studies, saying something like, “I have too much to do and can’t let my grades slip,” to explain your reclusive behaviour. With interventions: If suggested to have fluids intravenously (for low BP), ask “will that affect my weight?”; if advised to have a sandwich/biscuits (for low blood glucose level), ask about calorie content or if you really need to as you’re not hungry at present. Ending the scenario: The scenario will end when you are officially admitted to the hospital, and treatment has been initiated. You will display a gradual acceptance of your situation as healthcare staff discuss the next steps with you and hand over your case to the medical team. Show signs of fatigue and a hint of relief at the prospect of receiving help but remain reserved as the new team is introduced.
Student pre-brief psychological safety, learning outcomes, ground rules, orientation to simulation environment, students expectations, all given in group pre-simulation pre-brief	To all: Eddie Greene is a 20-year-old university student studying physiotherapy who fainted in the shared kitchen on the ward and has been brought to the Emergency Department. They are anxious and appear distressed, experiencing fatigue and mild dizziness. Scenario pre-brief: Nursing student: You are a newly graduated nurse working in the emergency department and have been asked to assess Eddie Greene, a 20-year-old physiotherapy student who fainted on the ward. They have been triaged, and bloods were taken, and an ECG was done. Scenario pre-brief: Medical student: You are an F1 doctor reviewing Eddie Greene, a 20-year-old university student who fainted. Please conduct a thorough assessment and collaborate with the nursing staff to address any immediate medical concerns.
HCA brief	You are the HCA working in the Emergency Department where Eddie Greene, a 20-year-old university student, has been brought in after fainting in the shared kitchen. Eddie is anxious and appears distressed, and you have been attempting to manage them. Scenario set-up: When the nursing staff arrives, greet them by explaining that Eddie has been "very anxious and hard to engage." You haven’t been able to gather much information from them about the fainting incident or their current health status. Starting position: Eddie may be sitting on the examination bed, appearing withdrawn or restless. You have just tried to talk to them, but they have provided minimal information about what happened. During the scenario: When the nurses arrive, provide a brief summary of your interactions with Eddie: “Eddie has been really anxious and hasn’t shared much about why they fainted. I’ve asked them a few times, but they seem really uneasy.” “They might feel more comfortable discussing things with you.” After providing your observations, step out of the room to give the nurses space to assess Eddie independently, but let them know you are available if needed: “They did bloods in Triage and an ECG, the ECG is here and we are awaiting the results.” “I’ll step out for a moment, but I’m here if you need anything.” Return as the medical students enter the scenario and say “Biochemistry has called with a critical result, the potassium is 2.2 mmol/L (3.5-5.0), magnesium is 1.6 mg/dL (1.8-2.4), and phosphorous is 2.5 mg/dL (2.7-4.5)." The results are on the computer. Key phrases to use: “They've been really anxious; I couldn’t get much information from them.” “Eddie seems uneasy. Maybe they’ll open up to you." Provide ECG paperwork. Provide blood work as prompted. Apply cannulation pad when cannulation requested or prompt if cued by offering to cannulate. “Would you like me to insert a cannula for you?”
Participant roles	x2 Student nurses assume the role of a newly qualified graduate, x2 medical students assume the role of an F1
Observer roles	Communication/handover information (Situation Background Assessment Recommendation (SBARR))/assessment skills/teamworking
Faculty information appropriate suggestions are incorporated to increase or decrease the scenario complexity	None
Debrief	
Plan for debrief	Co-debrief: Nursing Faculty and Lead Medical Faculty in the classroom where SMOTS is observed from
Debrief	Which debrief tool will you use? Debriefing questions you might want to help structure the debrief. Diamond Tool: Examples: Give a brief summary of what happened. What were the challenges? What worked well? If you were to do this again, how would you handle the situation differently?) Space for notes to make to bring up in debrief, key learning points. How to identify and address key performance gaps. Review of learning outcomes. How will observers be incorporated? Observers on SMOTS with areas to observe: Communication, handover information (SBARR), assessment skills/teamworking.
Post-simulation learning activities (see some examples below)	
Reflection	Identify any reflective activity and requirements
Skills lab	Follow-up skills lab work
Goal setting	Participants develop goals for future development
Self-assessment activity	Participants may do a post self-assessment activity
Quiz	Develop a quiz post-simulation to identify learning and achievement of learning outcomes

**Table 6:** Scenario 3: Elder Abuse and Carer Fatigue A-E: medical assessment, SMOTS: Scotia Medial Observation and Training System, QDS: four times a day, HCA: healthcare assistant, L-MCA: left middle cerebral artery.

Scenario overview	
Scenario synopsis	Stuart James is an 80-year-old male who presents to the Urgent Care Centre (UCC) for a wound review, following an injury he had sustained a few days earlier that was treated in the Emergency Department (ED). On arrival, Stuart is wearing long sleeves and long trousers despite it currently being fairly warm outside. He mobilises with a stick indoors but uses a wheelchair for longer distances. He arrived at the urgent care centre via wheelchair today. Stuart’s wife (Diane) places herself close to Stuart and dominates the conversations. She insists that Stuart just required the dressing once and she feels confident in doing it subsequently, as she is his full time carer. Stuart is very quiet and withdrawn and avoids eye contact initially. He is able to answer direct questions about his symptoms but is very quickly interrupted by Diane. The history that Diane provides is that three days ago, Stuart fell over and knocked himself on the corner of the TV stand leading to a fairly deep injury requiring sutures at ED. He has done this multiple times before but states that he is very clumsy and "bumps into things all the time." He has been slightly off his feet since being unwell and therefore has found it difficult to bring him to the General Practitioner (GP). She is his full time carer and therefore finds it difficult to manage all this. There are no other specific symptoms in the history. Stuart’s past medical history is that he had a left mid-cerebral artery (L-MCA) stroke three years ago. Although he has been able to rehabilitate well, he still has significant remaining impairments. This includes a right facial droop, mild dysarthria, persistent right-sided upper limb, and lower limb spasticity and weakness. There is no impairment on his left side. Stuart and Diane have three children. The eldest daughter lives in Sweden with her three children. She works as a bank manager and is often very busy so struggles to visit often although is able to call them every day. The second eldest is a son who is an artist in Edinburgh who often works long hours and nights and therefore does not contact them often. The youngest child also lives far in North Wales but visits them every few months. On assessment in the UCC, there is a fairly simple medical requirement by the nurse to clean and dress the leg wound. However, the team needs to continue to delve further into the slightly odd reactions from the wife and whether there is something else to consider. The healthcare team must navigate trying to get Stuart alone so he can discuss the issues at home without Diane there. Once the nursing students get Stuart alone he discloses that he has felt unsafe at home at times, whereby Diane has often lashed out and hit him. He has bruises of varying ages on his arms and legs. He is unsure on whether she does it on purpose but has felt that it often does happen when Diane is frustrated. He is worried as he does not want to get Diane in trouble and is also worried that he would not have anyone to look after him if they were separated. The nursing students should navigate encouraging Stuart to open up, involving the medical team and consequently involving the safeguarding teams.
Learner participants and learner level	Student, nurses in third year and final-year medical student, or foundation doctor (F1)
Scenario authors and content expertise	Dr Shreiya Narayanan, CTF, GWH; Dr Laura Sevenoaks, CTF, GWH; Dr Sung Yeon Kwak, CTF, GWH; Helen Krawczyk, Lecturer, Adult Nursing, OBU; Janice Watson, Senior Lecturer, Simulation and Immersive Technology, OBU; and Dr Jon Freeman, Clinical Lead and Psychologist, GWH
Up-to-date key references for scenario content	Adult safeguarding processes and guidelines. Mental State Examination. Mental Capacity Act. Care Act 2014
Estimated scenario time	10-15 minutes
Estimated debrief time	30-45 minutes
Curriculum/module/scenario learning outcomes	
Learning objectives	Knowledge: Able to identify physical and behavioural clues of abuse (e.g., unexplained bruises, anxiety in the patient when the spouse is around, signs of neglect, withdrawal, or inconsistent history); and able to identify and use appropriate screening tools for elder abuse. Skills: Conducts a thorough history and physical exam to differentiate between potential medical causes for the patient’s symptoms and non-medical issues such as abuse; demonstrates effective, sensitive communication with elderly patients, including how to ask about home life and safety in a non-threatening manner; demonstrates effective collaboration and team working with the interdisciplinary team to ensure the patient's safety, document findings, and initiate appropriate interventions such as reporting to social services or arranging follow-up care; and demonstrates an effective handover of clinical information using a structured format (Situation Background Assessment Recommendation (SBARR)). Attitudes/behaviours: Demonstrates effective communication and teamwork between different professional groups; demonstrates effective timely communication, empathy, support, and active listening with the patient and family if present; and addresses ethical considerations, including respecting the patient's autonomy while ensuring their protection, handling family dynamics, and managing potential fear or reluctance of the patient to disclose abuse.
Prerequisite knowledge required and/or pre-session work?	Adult safeguarding processes and guidelines. Mental State Examination. Mental Capacity Act
Scenario design	
Scenario environment	Location/setting: Urgent Care Centre. Patient: Actor. Monitor: Yes (to display observations). Props/equipment/medical supplies: Plinth, with linen; wheelchair; patient table; patient chair positioned beside the bed; blood pressure cuff, pulse oximeter, Dynamap; thermometer; spare trolley; and tray for med prep A-E trolley with nasal cannula/masks, needles, syringes, blood bottles and gas syringes. IV fluids various, giving sets syringe for fluid administration and medication administration. Capillary blood glucose/ketone meter. Simple dressings tray containing: Dressing pack, Normosol, Mepore dressing suitable size for wound, gauze, sterile gloves a range of sizes. Flucloxacillin capsule 1 g PO QDS, Paracetamol 500 mg tablets (x2). Make up/moulage/dress: Long-sleeved clothes. Simple moulage on left leg with wound which has been treated with stitches, simple Mepore dressing placed on top with discharge evident on dressing, erythema around wound edges. Multiple old and new bruises to limbs. Right shin wound-healing scar, erythema around the scar. Right forearm, mild swelling, and new bruise. Multimedia/paperwork: Patient observation chart (NEWS 2 chart) types of wounds and their management poster, if applicable. Medication chart for pain relief. Wound assessment chart. Audio/visual support: SMOTS, telephone, headset to HCA/plant, walkie talkies x2. Patient monitor for observations. People: Lead sim faculty (facilitator guiding the simulation). Actor as the patient (Stuart James). Patient’s life (Diane James). Nursing students (perform initial assessment). Medical students (called after initial nursing assessment). Healthcare Assistant (HCA) hands over to nursing student and expresses their concerns.
Client profile clinically realistic and summarised with detail regarding their characteristics, history and current status	Demographics: Stuart James. Age: 80 years. Address: 20 Lavender Street, Bristol, BS1 1AB. Weight: 70 kg. Height: 167 cm. Past medical history (PMH): Stuart had a L-MCA stroke two2 years ago which has left him with right facial droop, mild dysarthria, persistent right-sided upper limb and lower limb spasticity and weakness. There is no impairment on his left side. He suffers from heart failure, hypertension, and high cholesterol managed with medications. Family history (FH): Stuart's father died from a stroke at age 75, and his mother died aged 80 having had bowel cancer for a number of years. He has no siblings. Social history (SH): Stuart lives with his wife Diane in a three bedroomed semi-detached house. Stuart has a bed downstairs in the converted dining room, and they have a downstairs bathroom. They have three adult children that all live a moderate distance away and visit occasionally. Stuart is a previous smoker but has not smoked for 10 years and gave up alcohol when he gave up cigarettes. They are both retired. Stuart used to be in middle management, and Diane was a personal assistant in a large firm. Drug history (DH): Amlodipine 5 mg once daily in the morning (OM); Atorvastatin 80 mg once daily at night (ON); Bisoprolol 5 mg OM; Clopidogrel 75 mg OM; Ramipril 5 mg OM. Allergies: Cyclizine.
Initial Sim man setup or actor information for faculty	Provide all information here on initial settings required for your scenario. Mr. Stuart James. Dress: Stuart should be dressed in comfortable, slightly worn clothing appropriate for an elderly man; wearing long sleeves and long trousers despite it currently being fairly warm outside (e.g., cardigan, button-down shirt, loose pants, and supportive shoes). His appearance should be neat but understated, reflecting his age and dependency on his spouse for daily care. Wound simulation: A small, poorly healing wound should be applied to Stuart’s right shin. It should look like a superficial skin tear or scrape, surrounded by light redness that suggests it’s been there for a few days and may be surrounding cellulitis. Body language and positioning: Posture: Stuart should sit or stand in a slightly stooped posture, holding his forearm protectively or keeping it close to his body, suggesting that he’s either in pain or guarding the wound from further injury. Facial expression: He should appear anxious or withdrawn, with moments of hesitation or discomfort, particularly when his spouse is speaking or touching him. Interaction with spouse: Stuart avoids eye contact with Diane, showing subtle fear or discomfort, especially when she talks over him. When alone, he may sit more quietly, looking down or glancing at the door nervously. Observations (all within normal range). A: Patent. B: Respiratory rate 18 breaths per minute, clear on auscultation. C: Heart rate 75 beats per minute regular. Sats 99% in room air, blood pressure 120/80 mmHg. E: Temperature 37.8^o^C . Mrs. Diane James. Physical appearance and Props: Dress: Diane should be dressed neatly, in age-appropriate attire like a cardigan, blouse, and slacks. She should appear well put-together, to reflect a person who outwardly seems to be in control and taking good care of her husband. Accessories: A handbag or purse with medication bottles inside, which she may refer to during the simulation as part of her narrative about taking care of Stuart’s needs. Body language and positioning: Posture: Diane should sit or stand close to Stuart, often reaching over to touch his arm or speak over him, reinforcing her control over the conversation. She remains upright and confident, asserting dominance in the situation. Facial expression: She should maintain a pleasant, calm demeanour outwardly but should show signs of frustration or defensiveness when the conversation starts to focus on Stuart’s injuries or home life. Her facial expressions may turn slightly sharper if pressed with difficult questions. Interaction with Stuart: Diane will frequently interrupt or talk over Stuart, speaking for him or downplaying his symptoms. She may use gentle but patronizing touches (e.g., patting his hand or shoulder) to reinforce the image of being caring, while subtly maintaining control.
Actor brief	Role 1: Stuart James. Character overview: Mr. Stuart James is an 80-year-old man presenting with a small, poorly healing wound leg following an Emergency Department visit a few days ago. The wound is a subtle sign of physical abuse by his spouse, but he is reluctant to reveal this. His demeanour is quiet, anxious, and somewhat withdrawn. He avoids confrontation and is hesitant to discuss his injuries, especially when his spouse is present. Key Traits: Quiet and hesitant: Stuart answers questions vaguely and does not volunteer much information, especially when his spouse is in the room. Nervous and anxious: He appears visibly uncomfortable when his spouse is around, avoiding eye contact and showing subtle signs of distress, such as fidgeting or holding his wounded forearm protectively. Minimises the injury: Stuart downplays the wound, claiming it was a minor accident, such as "I must have scratched myself on something." He seems unsure about the details of how it happened. Subtle fear: When asked about his home life or if he feels safe, Stuart gives non-committal answers like “It’s fine” or “I don’t want to cause any trouble.” If gently pressed in private, he may allude to feeling unsafe but will not directly accuse his spouse, “It was just a little accident” “I was rushed”. Key Scenes: Initial Interaction: Stuart arrives with his spouse, who dominates the conversation. He seems passive and avoids giving specific details about how he got the wound. Physical Exam: The wound on his left leg is small but not healing well, and the skin around it is bruised and red and some slight oozing from the sutures. He flinches slightly when the area is touched and gives a vague explanation like, “It’s nothing, just a scrape.” Private Conversation: When questioned privately, he hesitates and then quietly says, “I don’t want to cause any problems. She’s just trying to help.” He might hint at feeling pressured or unsafe but will remain non-specific unless the students show empathy and patience. Role 2: Mrs. Diane James Character Overview: Mrs. Diane James is a 75-year-old woman who appears caring but is subtly controlling and dismissive of her husband’s health concerns. She downplays his injury and takes control of the conversation, trying to prevent Stuart from revealing too much about their home life. She provides inconsistent explanations for the wound, becoming slightly defensive if questioned too closely. Key Traits: Controlling: Diane often interrupts Stuart or speaks for him, making excuses for his injury and minimising its seriousness. She will say things like, “Oh, it’s just a small cut,” or “He’s always so careless with things.” Inconsistent explanations: When asked how Stuart got the wound, she might change her story (e.g., “He must’ve scratched himself on something,” “I think it happened in the garden”). If pressed, she becomes more defensive, saying, “He’s fine, really.” Defensive: If students ask directly about their home life or John’s safety, she might become defensive and insist, “I’m the one taking care of him, and he’s just confused,” or “He’s always been a little clumsy, nothing to worry about.” Dismissive: Diane downplays the wound’s significance and shifts attention away from it by saying, “It’s healing fine” or “He just doesn’t look after himself as well as he should.” Key Scenes: Initial Interaction: Diane speaks over Stuart, answering most of the questions and providing excuses for the wound, like “Oh, he’s always getting little cuts like that. Nothing serious.” Defensive Behaviour: When students ask for more details about how the wound occurred or Stuart’s general health, Diane becomes slightly defensive, deflecting attention and insisting that there’s nothing wrong, “We’re just here for a wound re-dressing and let me take Stuart home” Reluctance to Leave: If students ask to speak to Stuart privately, Diane may resist, saying, “I need to be here in case he forgets something,” or “He can’t remember things well on his own.” If they do this sensitively or by asking if you want to have a chat and a cup of tea go along with it and leave Stuart alone with the nurse/Dr. If they do not do this successfully the Healthcare Assistant will come in and ask if you could come along to reception as they have an important question about Stuart's medication at which point you leave. You may return so be prepared to re-enter the scenario if they decide they want to talk through next steps with you both. If they do this and disclose their concerns about the injuries, be upset and look crestfallen, admitting that it is “just too much” The scenario will end at this point if not earlier. General Instructions for Both Actors: Realistic Timing: Avoid revealing too much too quickly. Let the students ask probing questions and take note of your behaviours. Emotional Tension: Keep emotions controlled—focus on subtle displays of fear (for Stuart) and control (for Diane), rather than overt displays of aggression or anxiety. Adapting: Respond naturally to the students’ questions and actions, adjusting your responses based on their approach while staying true to your character’s motivations.
Student pre-brief psychological safety, learning outcomes, ground rules, orientation to simulation environment, students expectations, all given in group pre simulation pre-brief	Scenario pre-brief: Nursing student. You are a newly graduated nurse working in Urgent Care Centre. Your next patient is Stuart James, an 80-year-old male who presents for a wound review with his wife. Please review this patient. Scenario pre-brief: Medical Student. You are a Foundation Year 1 doctor rotating to Urgent Care Centre. You have been asked by the nurse to review Stuart James, an 80-year-old male who has presented for a wound review. Please receive a handover from the nurse and review the patient.
HCA brief	You are the HCA working in the Practice who is helping the nurse review. Mr Stuart James. Scenario set-up: When the nursing staff arrives, greet them by explaining that Mr James has come in with his wife for a wound review. You don’t know any further information at present. During the scenario: Initial Interaction with the patient (Mr. Stuart James): When you first approach Mr James, you will assist with basic tasks like helping him get comfortable, taking his vital signs, or offering support with mobility. Be gentle with his right leg where the wound is present. When handling his arm, you should notice his discomfort and subtly comment, "Are you okay there, Mr. James? It seems a little sore," which might prompt the students to explore further. Observe any bruises or injuries that don’t align with his explanation of the cause (e.g., "I scratched myself gardening"). You can make innocent comments to the students like, "I noticed a few bruises—did you mention he's had some falls lately?" Interaction with the spouse (Mrs. Diane James ): When Mrs. James is present, she might try to dominate the conversation or answer questions for Mr. James. Stay neutral.
Participant roles	x2 Student nurses assume the role of a newly qualified graduate, x1 medical students assume the role of an F1
Observer roles	Communication/handover information (Situation Background Assessment Recommendation (SBARR))/assessment skills/teamworking
Faculty information Appropriate suggestions are incorporated to increase or decrease the scenario complexity	Would also consider domestic abuse pathways, care and support needs and carers assessment/needs for support under Care Act 2014. Also considerations of if wound/injury is consistent with story
Debrief	
Plan for debrief	Co-debrief: Nursing Faculty and Lead Medical Faculty and Safeguarding Lead in the classroom where SMOTS is observed from.
Debrief	Which debrief tool will you use? Debriefing questions you might want to help structure the debrief. Pause technique-see case flow
Post-simulation learning activities (see some examples below)	
Reflection	Identify any reflective activity and requirements
Skills lab	Follow-up skills lab work
Goal setting	Participants develop goals for future development
Self-assessment activity	Participants may do a post self-assessment activity
Quiz	Develop a quiz post-simulation to identify learning and achievement of learning outcomes

Appendix 2: Questionnaire 

Appendix 3: Questionnaire raw data

**Table 7:** Pre-Session Questionnaire Raw Data

Pre-session	I understand common mental conditions and their impacts on patients	I feel confident in communicating with patients experiencing mental health issues	I feel prepared to respond to a mental health crisis in a clinical setting	I understand the roles of other health professionals in the care of patients with mental health needs	I value the contribution of different healthcare disciplines in managing health conditions	I feel confident collaborating with students from other health professions	Interprofessional teamwork is essential for effective mental health care delivery
5-Strongly agree	4	0	0	1	12	2	20
4-Agree	11	7	3	10	11	13	6
3-Neither agree nor disagree	12	12	14	15	6	11	2
2-Disagree	2	9	9	3	0	3	1
1-Strongly disagree	0	1	3	0	0	0	0
Total	29	29	29	29	29	29	29

**Table 8:** Post-Session Questionnaire Raw Data

Post-session	I understand common mental conditions and their impacts on patients	I feel confident in communicating with patients experiencing mental health issues	I feel prepared to respond to a mental health crisis in a clinical setting	I understand the roles of other health professionals in the care of patients with mental health needs	I value the contribution of different healthcare disciplines in managing health conditions	I feel confident collaborating with students from other health professions	Interprofessional teamwork is essential for effective mental health care delivery
5-Strongly agree	11	11	7	10	22	20	27
4-Agree	14	12	17	17	6	7	1
3-Neither agree nor disagree	3	5	4	1	0	1	0
2-Disagree	0	0	0	0	0	0	0
1-Strongly disagree	0	0	0	0	0	0	0
Total	28	28	28	28	28	28	28

## References

[1] (2025). Centre for the Advancement of Interprofessional Education: Interprofessional education: a definition. http://www.caipe.org.uk.

[2] Buring SM, Bhushan A, Broeseker A, Conway S, Duncan-Hewitt W, Hansen L, Westberg S (2009). Interprofessional education: definitions, student competencies, and guidelines for implementation. Am J Pharm Educ.

[3] Barr H, Freeth D, Hammick M (2000). Evaluations of Interprofessional Education: A United Kingdom Review for Health and Social Care. Centre for the Advancement of Interprofessional Education in Primary Health and Community Care.

[4] Lumague M, Morgan A, Mak D (2006). Interprofessional education: the student perspective. J Interprof Care.

[5] Reeves S, van Schaik S (2012). Simulation: a panacea for interprofessional learning?. J Interprof Care.

[6] van Soeren M, Devlin-Cop S, Macmillan K, Baker L, Egan-Lee E, Reeves S (2011). Simulated interprofessional education: an analysis of teaching and learning processes. J Interprof Care.

[7] Rauen CA (2004). Simulation as a teaching strategy for nursing education and orientation in cardiac surgery. Crit Care Nurse.

[8] Issenberg SB, McGaghie WC, Petrusa ER, Lee Gordon D, Scalese RJ (2005). Features and uses of high-fidelity medical simulations that lead to effective learning: a BEME systematic review. Med Teach.

[9] Bada SO, Olusegun S (2015). Constructivism learning theory: a paradigm for teaching and learning. J Res Method Educ.

[10] Motola I, Devine LA, Chung HS, Sullivan JE, Issenberg SB (2013). Simulation in healthcare education: a best evidence practical guide. AMEE Guide No. 82. Med Teach.

[11] Zendejas B, Brydges R, Wang AT, Cook DA (2013). Patient outcomes in simulation-based medical education: a systematic review. J Gen Intern Med.

[12] Piette AE, Attoe C, Humphreys R, Cross S, Kowalski C (2018). Interprofessional simulation training for community mental health teams: findings from a mixed methods study. J Interprof Care.

[13] Barr H, Koppel I, Reeves S, Hammick M, Freeth D (2005). Effective Interprofessional Education: Argument, Assumption and Evidence.

[14] Zechariah S, Ansa BE, Johnson SW, Gates AM, Leo G (2019). Interprofessional education and collaboration in healthcare: an exploratory study of the perspectives of medical students in the United States. Healthcare (Basel).

[15] Clarke V, Braun V (2013). Teaching thematic analysis: overcoming challenges and developing strategies for effective learning. Psychol.

[16] Langton V, Dounas D, Moore A, Bacchi S, Thomas J (2021). The use of interprofessional simulation interventions in medical student education: a scoping review. Focus Health Prof Educ.

[17] Hamilton P, Coey-Niebel C, McCaig J (2021). Evaluation of Inter-Professional Education (IPE) with medical, nursing and pharmacy students through a simulated IPL Educational Intervention. Int J Clin Pract.

[18] Attoe C, Lavelle M, Sherwali S, Rimes K, Jabur Z (2019). Student interprofessional mental health simulation (SIMHS): evaluating the impact on medical and nursing students, and clinical psychology trainees. J Ment Health Train Educ Pract.

[19] Naismith LM, Kowalski C, Soklaridis S, Kelly A, Walsh CM (2020). Participant perspectives on the contributions of physical, psychological, and sociological fidelity to learning in interprofessional mental health simulation. Simul Healthc.

